# Supplementary figures and images for: Endogenous PGD2 acting on DP2 receptor counter regulates Schistosoma mansoni infection-driven hepatic granulomatous fibrosis
Source: PLoS Pathog. 2024 Aug 22;20(8):e1011812. doi: 10.1371/journal.ppat.1011812 (PMC11386465; doi:10.1371/journal.ppat.1011812)

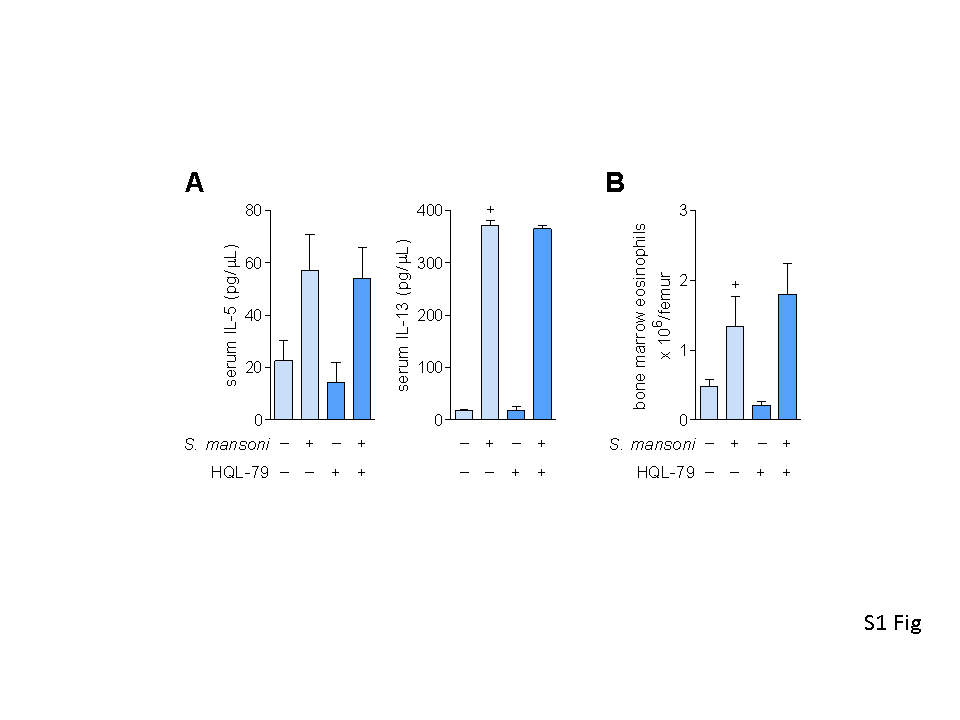

Supplement: S1 Fig — A shows serum levels of IL-5 and IL-13. B shows eosinophil numbers found at bone marrow. Values are expressed as the mean ± SEM from at least 5 animals per group (experiment was repeated at least once). +p < 0.05 compared to non-infected control group. (TIF) [file ppat.1011812.s001.TIF]

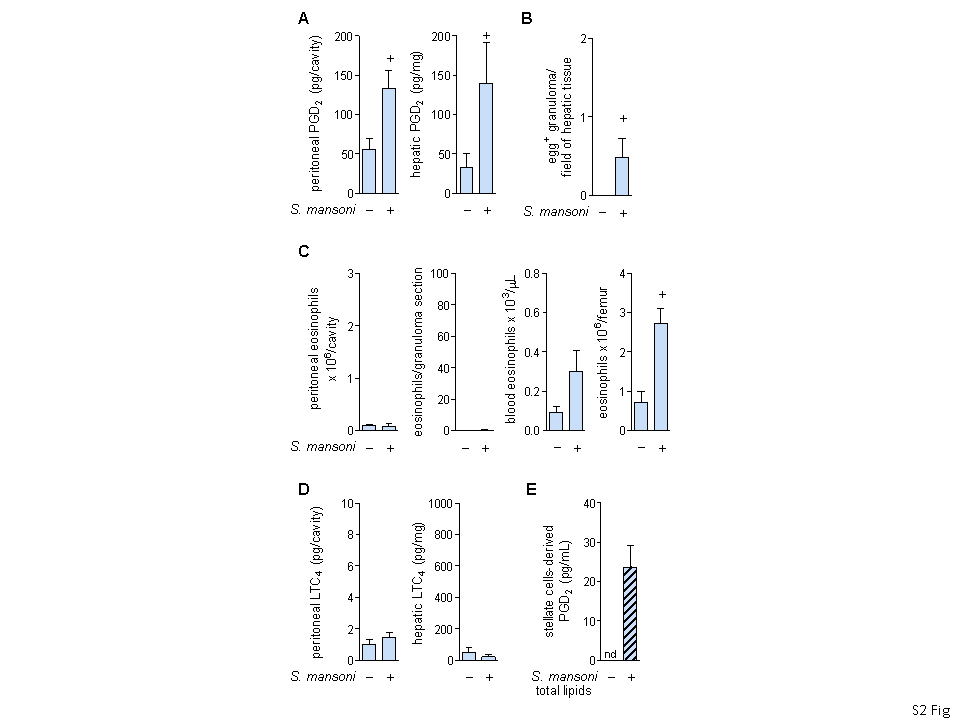

Supplement: S2 Fig — Peritoneal lavages and livers were collected 6 wpi with S. mansoni cercariae. A shows PGD2 amounts detected by specific EIA kit in cell-free peritoneal fluids and in liver homogenates. In B, total numbers of egg-encasing hepatic granulomas are shown. C shows eosinophil numbers found in either peritoneal compartment, hepatic granuloma, peripheral blood, or bone marrow space. D shows peritoneal and liver LTC4 levels. Values are expressed as the mean ± SEM from at least 5 animals per group. +p < 0.05 compared to non-infected control group. In E, hepatic granuloma-derived stellate cells were isolated 6 wpi from S. mansoni-infected mice and then stimulated in vitro for 2 h with total lipids extracted from isolated parasites; PGD2 synthesis was quantified in cell-free supernatants. Values are expressed as the mean ± SEM from 2 preparations of hepatic granuloma-derived stellate cells. (TIF) [file ppat.1011812.s002.TIF]

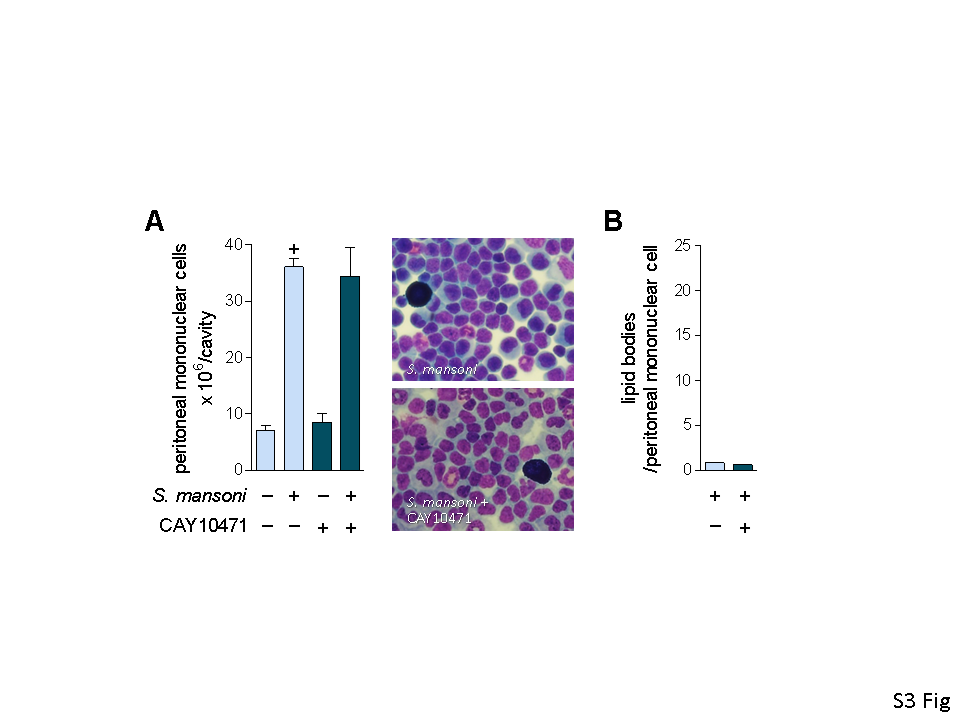

Supplement: S3 Fig — A shows numbers of mononuclear cells found at peritoneal space as well as representative images of peritoneal cells 8 wpi of S. mansoni-infected mice (top image) and in mice treated with CAY10471 and S. mansoni infected (bottom image). B shows numbers of cytoplasmic lipid body organelles found in peritoneal mononuclear cells. Values are expressed as the mean ± SEM from at least 5 animals per group (experiment was repeated at least once). +p < 0.05 compared to non-infected control group. (TIF) [file ppat.1011812.s003.TIF]

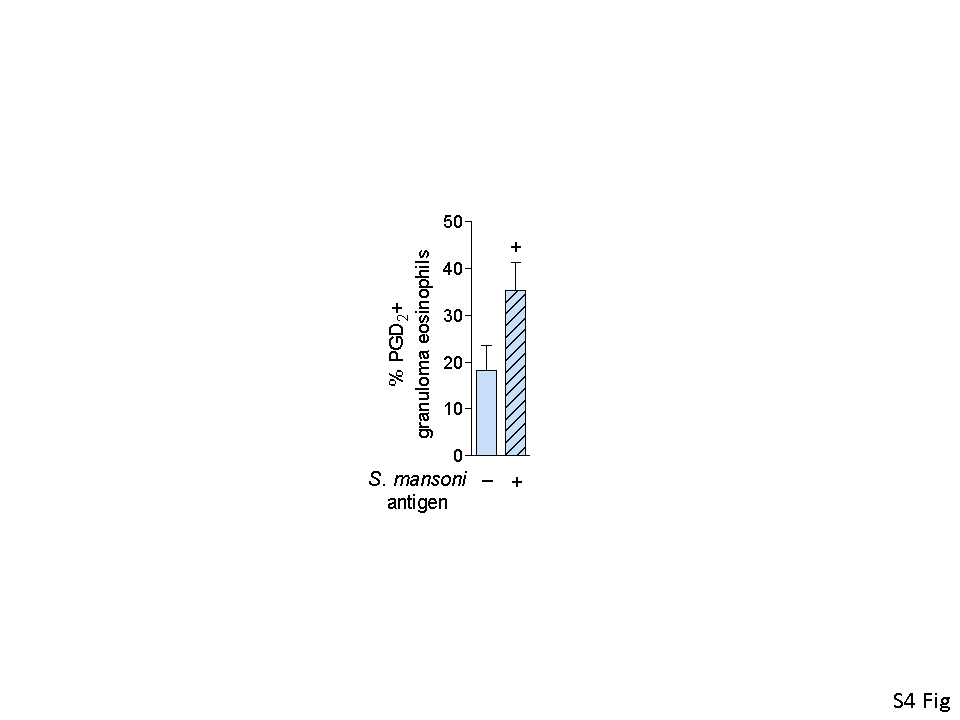

Supplement: S4 Fig — Eosinophils were isolated from 8 wpi schistosomal hepatic granulomas (purity about 90%) and stimulated in vitro for 1 h with S. mansoni antigen (0.5 μg/mL). Percentage of in vitro stimulated eosinophils (as indicated) exhibiting cytoplasmic immunostaining for LTC4 (LTC4+ eosinophils) under fluorescence microscopy in Eicosacell preparations are shown. Values are expressed as the mean ± SEM from 3 preparations of granuloma-isolated eosinophils. +p < 0.05 compared to non-stimulated eosinophils. (TIF) [file ppat.1011812.s004.TIF]
