# Supplementary material for: Endogenous PGD2 acting on DP2 receptor counter regulates Schistosoma mansoni infection-driven hepatic granulomatous fibrosis
Source: PLoS Pathog. 2024 Aug 22;20(8):e1011812. doi: 10.1371/journal.ppat.1011812 (PMC11386465; doi:10.1371/journal.ppat.1011812)
Supplement: S1 Table — S. mansoni infection in mice was achieved by active percutaneous penetration by 60 cercariae. Osmotic pumps containing a 0.1% DMSO solution were subcutaneously implanted (3.5 wpi) in both non-infected and S. mansoni-infected animals (columns labelled “DMSO“). Animals that were not implanted with subcutaneous pumps were used as controls (columns labelled “-“). All animals were alive at 8 wpi, when they were euthanized. Livers of all non-infected animals were normal, showing no macroscopic or histopathological alterations. Individual values and mean ± SEM from 2 or 3 animals per group are displayed. (DOCX) [file ppat.1011812.s005.docx]

**S1 Table**. Lack of impact of 0.1% DMSO solution (the vehicle solution employed for HQL-79, CAY10471 or MK571 treatments delivered by osmotic pumps) on non-infected or *S. mansoni*-infected animals.*^a^*

| **blood eosinophils x 10^3^/µL** | | | | **peritoneal eosinophils x 10^6^ /cavity** | | | | **egg^+^ granuloma/field of hepatic tissue** | | | |
| --- | --- | --- | --- | --- | --- | --- | --- | --- | --- | --- | --- |
| non-infected | | *S. mansoni* | | non-infected | | *S. mansoni* | | non-infected | | *S. mansoni* | |
| - | DMSO | - | DMSO | - | DMSO | - | DMSO | - | DMSO | - | DMSO |
| 0.1 | 0.2 | 0.3 | 0.3 | 0.2 | 0.2 | 1.1 | 0.9 | 0.0 | 0.0 | 1.2 | 0.9 |
| 0.2 | 0.1 | 0.3 | 0.4 | 0.3 | 0.2 | 0.8 | 0.6 | 0.0 | 0.0 | 0.5 | 0.6 |
| 0.1 |  | 0.2 |  | 0.1 |  | 0.5 |  | 0.0 |  | 0.9 |  |
| 0,13 ± 0.03 | 0,15 ± 0.05 | 0,27 ± 0.03 | 0,35 ± 0.05 | 0,20 ± 0.06 | 0,20 ± 0.00 | 0,80 ± 0.17 | 0,75 ± 0.15 | 0,00 ± 0.00 | 0,00 ± 0.00 | 0,87 ± 0.20 | 0,75 ± 0.15 |

*^a^* *S. mansoni* infection in mice was achieved by active percutaneous penetration by 60 cercariae. Osmotic pumps containing a 0.1% DMSO solution were subcutaneously implanted (3.5 wpi) in both non-infected and *S. mansoni*-infected animals (columns labelled “DMSO“). Animals that were not implanted with subcutaneous pumps were used as controls (columns labelled “-“). All animals were alive at 8 wpi, when they were euthanized. Livers of all non-infected animals were normal, showing no macroscopic or histopathological alterations. Individual values and mean ± SEM from 2 or 3 animals *per* group are displayed.
