# Supplementary material for: Endogenous PGD2 acting on DP2 receptor counter regulates Schistosoma mansoni infection-driven hepatic granulomatous fibrosis
Source: PLoS Pathog. 2024 Aug 22;20(8):e1011812. doi: 10.1371/journal.ppat.1011812 (PMC11386465; doi:10.1371/journal.ppat.1011812)
Supplement: S2 Table — The data for each group presented in the manuscript is listed in individual tables for each graph indicated by Figure number and letter. Mean ± SEM for each group is provided in the bottom line. (DOCX) [file ppat.1011812.s006.docx]

| **Figure 1B** | | | | |
| --- | --- | --- | --- | --- |
| Number of eggs/g of feces | | | | |
| 3wpi | 4 wpi | 6 wpi | 8 wpi | 11 wpi |
| 0. | 0. | 48. | 600. | 2784. |
| 0. | 0. | 192. | 1896. | 48. |
| 0. | 0. | 0. | 1488. | 24. |
| 0. | 0. | 600. | 2136. | 720. |
| 0. | 0. | 24. | 240. | 72. |
| 0. | 0. | 0. | 120. |  |
|  | 0. | 1200. | 4128. |  |
|  | 0. |  | 3840. |  |
|  | 0. |  | 768. |  |
|  | 0. |  | 912. |  |
|  | 0. |  | 792. |  |
|  | 0. |  | 1560. |  |
|  | 0. |  |  |  |
| 0.0 ± 0.0 | 0.0 ± 0.0 | 295 ± 171 | 1540 ± 376 | 730 ± 530 |

| **Figure 1C** | | | | | | |
| --- | --- | --- | --- | --- | --- | --- |
| Peritoneal PGD2 (3 wpi) | |  | Peritoneal PGD2 (8wpi) | | | |
| Non Infected | Infected |  | Non Infected | Infected | NonInf+HQL | Inf + HQL |
| 77.72630 | 289.226600 |  | 79.87932 | 235.1907 | 104.5938 | 141.80270 |
| 125.55240 | 112.000200 |  | 83.91598 | 224.6978 | 127.4346 | 94.80009 |
| 93.33562 | 85.798500 |  | 115.45000 | 282.6447 | 145.0107 | 168.44830 |
| 126.22840 | 88.329210 |  | 143.03560 | 220.1744 | 186.8975 | 194.18400 |
| 101.85000 | 91.632600 |  |  | 326.3882 |  | 193.63180 |
| 103.00120 | 135.653300 |  |  |  |  |  |
| 104.6 ± 7.7 | 133.8 ± 32.0 |  | 105.6 ± 14.8 | 257.8 ± 20.4 | 141.0 ± 17.4 | 158.6 ± 18.6 |

| **Figure 1D** | | | | | | |
| --- | --- | --- | --- | --- | --- | --- |
| Hepatic PGD2 (3 wpi) | |  | Hepatic PGD2 (8wpi) | | | |
| Non infected | Infected |  | Non Infected | Infected | NonInf+HQL | Inf + HQL |
| 57.99550 | 53.25691 |  | 38.265800 | 268.944800 | 31.077780 | 75.913230 |
| 155.23660 | 89.32660 |  | 0.000000 | 144.533100 | 0.000000 | 51.670610 |
| 48.03215 | 45.39220 |  | 0.000000 | 117.173800 | 69.687490 | 0.000000 |
| 69.32650 | 61.02215 |  | 90.859310 | 167.602100 | 0.000000 | 0.000000 |
| 70.33210 | 47.62530 |  | 72.179360 | 73.374050 | 0.000000 | 130.304900 |
| 49.12030 |  |  |  |  |  |  |
| 75.0 ± 16.5 | 59.3 ± 8.0 |  | 40.3 ± 18.5 | 154.3 ± 32.7 | 20.2 ± 13.8 | 51.6 ± 24.6 |

| **cont. Figure 1D** | | | |
| --- | --- | --- | --- |
| Hepatic PGE2 (8wpi) | | | |
| Non Infected | Infected | NonInf+HQL | Inf + HQL |
| 87.33365 | 124.81360 | 81.58754 | 113.77210 |
| 107.06750 | 125.22880 | 81.83960 | 148.23670 |
| 78.97285 | 75.12640 | 126.97550 | 144.26430 |
| 84.35777 | 115.23860 | 114.72180 | 85.41638 |
| 97.92657 | 135.05010 | 101.61850 | 121.76280 |
| 91.1 ± 5.0 | 115.1 ± 10.5 | 101.3 ± 9.0 | 122.7 ± 11.4 |

| **Figure 2A** | | | | | | |
| --- | --- | --- | --- | --- | --- | --- |
| OH-proline (ng/mg of hepatic tissue) | | | |  | Number of egg+ granulomas | |
| Non Infected | Infected | NonInf+HQL | Inf + HQL |  | Infected | Inf + HQL |
| 342.6 | 325.9 | 222.8 | 504.7 |  | 1.55 | 1.15 |
| 355.3 | 49.8 | 282.2 | 696.2 |  | 1.45 | 1.75 |
| 319.5 | 210.5 | 269.3 | 604.5 |  | 1.55 | 1.55 |
| 314.4 | 432.9 | 331.2 | 499.2 |  | 1.80 | 0.95 |
| 324.8 | 351.8 | 396.8 | 585.1 |  | 1.80 | 1.35 |
| 254.9 | 495.1 | 281.1 | 509.4 |  |  |  |
| 335.9 | 592.6 |  | 652.6 |  |  |  |
|  | 505.0 |  |  |  |  |  |
| 321,1 ± 12,2 | 370,5 ± 62,3 | 297,2 ± 24,4 | 578,8 ± 29,5 |  | 1.6 ± 0.1 | 1.4 ± 0.1 |

| **cont. Figure 2A** | | | | |
| --- | --- | --- | --- | --- |
| Granuloma area | |  | fluorescence/granuloma section | |
| Infected | Infected+HQL |  | Infected | Inf + HQL |
| 11.1416 | 12.5782 |  | 1.460 | 2.728460 |
| 9.8697 | 14.2502 |  | 0.063 | 3.353804 |
| 11.1974 | 11.1601 |  | 1.450 | 2.965200 |
| 10.7996 | 11.7943 |  | 0.100 | 3.540000 |
| 9.6301 | 18.2782 |  | 2.800 | 5.200000 |
| 10.5 ± 0.3 | 13.6 ± 1.3 |  | 1.2 ± 0.5 | 3.6 ± 0.4 |

| **Figure 2B** | | | | | | |  |
| --- | --- | --- | --- | --- | --- | --- | --- |
| OH-proline (ng/mg of hepatic tissue) | | | |  | Number of egg+ granulomas | | |
| Non Infected | Infected | NonInf+CAY | Inf + CAY |  | Infected | Inf + CAY | |
| 156.9687 | 494.5323 | 195.3569 | 722.1285 |  | 2.07 | 2.21 | |
| 185.7365 | 458.427 | 189.8576 | 552.7895 |  | 0.64 | 3.50 | |
| 292.437 | 394.0135 | 242.3092 | 539.6285 |  | 0.86 | 1.27 | |
| 174.7609 | 513.9855 | 267.5 | 750.835 |  | 1.38 | 0.09 | |
| 192.6066 | 391.1675 | 155.0 | 463.945 |  | 1.70 | 1.03 | |
|  |  |  |  |  | 1.48 | 0.79 | |
|  |  |  |  |  | 1.39 |  | |
|  |  |  |  |  | 0.52 |  | |
| 200,5 ± 23,7 | 450,4 ± 25,2 | 210,0 ± 19,9 | 605,9 ± 55,6 |  | 1.3 ± 0.2 | 1.5 ± 0.5 | |

| **cont. Figure 2B** | | | | |
| --- | --- | --- | --- | --- |
| Granuloma area | |  | fluorescence/granuloma section | |
| Infected | Infected+CAY |  | Infected | Inf + CAY |
| 9.29535 | 10.12500 |  | 1.727313 | 1.733398 |
| 11.13385 | 10.22423 |  | 1.803074 | 2.472290 |
| 11.00966 | 11.77716 |  | 0.920700 | 3.401209 |
| 10.37039 | 16.97679 |  | 1.499517 | 3.701074 |
| 7.97497 | 14.32553 |  | 1.923693 | 2.785400 |
| 11.25980 | 11.96342 |  | 1.305446 | 1.553589 |
| 11.04262 |  |  | 1.586635 |  |
| 11.53044 |  |  | 1.440263 |  |
| 10.5 ± 0.4 | 12.6 ± 1.1 |  | 1.5 ± 0.1 | 2.6 ± 0.4 |

| **Figure 2C** | | | | | | | | |
| --- | --- | --- | --- | --- | --- | --- | --- | --- |
| Hepatic TGFbeta (ng/mg) | | | |  | Hepatic IL-13 (ng/mg) | | | |
| Non Infected | Infected | NonInf+HQL | Inf + HQL |  | Non Infected | Infected | NonInf+HQL | Inf + HQL |
| 2.310571 | 5.490362 | 8.219016 | 12.91756 |  | 0.9656325 | 1.5993790 | 1.63220 | 2458.20100 |
| 3.230783 | 8.219016 | 3.517864 | 16.16917 |  | 0.4092365 | 1.9852370 | 0.9568461 | 2658.38600 |
| 4.014278 | 9.484702 | 4.759730 | 31.97748 |  | 1.0003270 | 2.0240730 | 1.2375420 | 5892.01900 |
| 0.000000 | 9.168071 | 5.158370 | 23.62439 |  | 0.3942789 | 4.7279620 | 0.9315560 | 4389.01300 |
| 0.000000 | 7.430095 | 0.000000 | 11.03590 |  | 0.5993256 | 1.9613260 | 1.0213790 | 3015.49300 |
| 0.000000 | 6.484390 | 0.000000 | 17.56266 |  | 1.1862360 |  | 0.6573891 | 1903.24900 |
| 1.6 ± 0.7 | 7.7 ± 0.6 | 3.6 ± 1.3 | 18.9 ± 3.1 |  | 0.8 ± 0.1 | 2.5 ± 0.6 | 1.1 ± 0.1 | 3.4 ± 0.6 |

| **Figure 2D** | | | | | | | | |
| --- | --- | --- | --- | --- | --- | --- | --- | --- |
| Hepatic TGFbeta (ng/mg) | | | |  | Hepatic IL-13 (ng/mg) | | | |
| Non Infected | Infected | NonInf+CAY | Inf + CAY |  | Non Infected | Infected | NonInf+CAY | Inf + CAY |
| 1.82160 | 13.72900 | 0.8385752 | 68.72100 |  | 1.435260 | 2.3991580 | 1.007094 | 2.018218 |
| 2.32700 | 15.82400 | 2.239421 | 84.76000 |  | 2.105073 | 0.8473176 | 1.113849 | 3.290404 |
| 3.35100 | 14.21800 | 0.6960676 | 128.31800 |  | 1.575081 | 1.7368190 | 1.672070 | 2.936672 |
|  | 19.10600 | 1.356971 | 76.10800 |  | 1.639722 | 1.8124500 | 1.392306 | 3.545994 |
|  | 17.60500 | 1.213681 | 28.09100 |  | 1.182450 | 2.1050730 | 1.942325 | 2.606993 |
|  | 26.55200 | 1.124235 | 111.59300 |  | 1.456750 | 2.6069930 |  | 4.297104 |
|  | 33.52100 |  |  |  | 1.747617 | 2.7166800 |  |  |
|  | 10.25100 |  |  |  |  | 2.6837510 |  |  |
| 2.5 ± 0.5 | 18.9 ± 2.7 | 1.3 ± 0.2 | 82.9 ± 14.3 |  | 1.6 ± 0.1 | 2.1 ± 0.2 | 1.4 ± 0.2 | 3.1 ± 0.3 |

| **Figure 3A** | | | | | | | | |
| --- | --- | --- | --- | --- | --- | --- | --- | --- |
| Blood eosinophils | | | |  | Peritoneal eosinophils | | | |
| Non Infected | Infected | NonInf+HQL | Inf + HQL |  | Non Infected | Infected | NonInf+HQL | Inf + HQL |
| 0.40 | 0.30 | 0.10 | 0.50 |  | 0.0565 | 1.7920 | 0.000 | 0.0000 |
| 0.20 | 0.40 | 0.10 | 0.10 |  | 0.0875 | 1.6120 | 0.000 | 0.0000 |
| 0.10 | 0.90 | 0.00 | 0.30 |  | 0.0895 | 1.2030 | 0.000 | 0.0000 |
| 0.00 | 1.20 | 0.20 | 0.10 |  | 0.0000 | 3.4000 | 0.090 | 0.4125 |
| 0.10 | 0.10 | 0.00 | 0.00 |  | 0.1140 | 1.4840 | 0.000 | 0.0000 |
| 0.00 |  | 0.20 |  |  | 0.0000 |  | 0.000 |  |
| 0.13 ± 0.06 | 0.58 ± 0.2 | 0.10 ± 0.4 | 0.20 ± 0.1 |  | 0.06 ± 0.02 | 1.90 ± 0.4 | 0.02 ± 0.02 | 0.08 ± 0.08 |

| **Figure 3B** | | | | |
| --- | --- | --- | --- | --- |
| Eosinophils/granuloma | |  | Hepatic LTC4/D4/E4 | |
| Infected | Infected+HQL |  | Infected | Inf + HQL |
| 370.1429 | 236.1250 |  | 1748.6880 | 186.8778 |
| 384.1429 | 165.1429 |  | 1084.9010 | 0.0000 |
| 267.7143 | 197.7143 |  | 645.9420 | 44.4065 |
| 237.5714 | 253.5714 |  | 615.6329 | 317.8710 |
| 373.0000 | 190.7500 |  | 1145.0780 | 147.2000 |
| 326.5 ± 31 | 208.7 ± 16 |  | 1048 ± 206 | 139 ± 56 |
| Obs. These are also the individual values plotted in the linear regression showed in **Figure 3C (bottom graph)** | | | | |

| **Figure 3C (top graph)** | | | | |
| --- | --- | --- | --- | --- |
| OH-proline | |  | Hepatic LTC4/D4/E4 | |
| Infected | Infected+HQL |  | Infected | Inf + HQL |
| 49.89505 | 696.194 |  | 1748.6880 | 186.8778 |
| 210.4695 | 604.5055 |  | 1084.9010 | 0.0000 |
| 432.9269 | 499.1975 |  | 645.9420 | 44.4065 |
| 351.8024 | 585.1325 |  | 615.6329 | 317.8710 |
| 495.0631 | 509.4445 |  | 1145.0780 | 147.2000 |
| Obs. These are the individual values plotted in the linear regression showed in **Figure 3C (top graph)** | | | | |

| **Figure 4A and Figure 4B** | | | | | | |
| --- | --- | --- | --- | --- | --- | --- |
| Peritoneal eosinophils | | | |  | Lipid bodies/eosinophils | |
| Non Infected | Infected | NonInf+CAY | Inf + CAY |  | Infected | Inf + CAY |
| 0.2 | 1.7 | 0.2 | 2.2 |  | 21.50 | 22.72 |
| 0.0 | 4.4 | 0.0 | 1.4 |  | 22.44 | 16.32 |
| 0.1 | 5.3 | 0.1 | 0.4 |  | 22.18 | 15.80 |
| 0.0 | 6.7 | 0.0 | 1.3 |  | 21.78 | 16.70 |
| 0.0 | 2.9 | 0.1 | 2.6 |  | 21.90 |  |
| 0.0 | 0.8 | 0.0 | 2.8 |  | 21.33 |  |
| 0.1 | 2.9 |  |  |  |  |  |
|  | 5.3 |  |  |  |  |  |
| 0.06 ± 0.03 | 3.8 ± 0.7 | 0.07 ± 0.03 | 1.8 ± 0.4 |  | 21.9 ± 0.2 | 17.9 ± 1.6 |

| **Figure 4D and Figure 4E** | | | | |
| --- | --- | --- | --- | --- |
| % LTC4+ peritoneal eosinophils | |  | Peritoneal LTC4/D4/E4 (ng/cavity) | |
| Infected | Inf + CAY |  | Infected | Inf + CAY |
| 87.0 | 61.6 |  | 7.177908 | 7.676992 |
| 90.0 | 50.0 |  | 14.144010 | 2.626500 |
| 66.6 | 51.0 |  | 2.404322 | 0.5534828 |
| 89.3 | 34.0 |  | 18.863310 | 0.4683588 |
| 54.8 | 33.3 |  | 3.897485 | 3.950918 |
| 73.3 | 36.6 |  | 21.960000 | 0.2271059 |
| 50.0 |  |  | 10.773430 |  |
| 60.0 |  |  | 5.033064 |  |
| 71.4 ± 5.7 | 44.4 ± 4.7 |  | 10.5 ± 2.6 | 2.6 ± 1.2 |

| **Figure 5A** | | | | |
| --- | --- | --- | --- | --- |
| Eosinophils/granuloma | |  | Hepatic LTC4/D4/E4 | |
| Infected | Infected+CAY |  | Infected | Inf + CAY |
| 416.5 | 228.5 |  | 1795.747000 | 1282.5880 |
| 458.0 | 218.0 |  | 1127.024000 | 1081.6530 |
| 659.0 | 253.5 |  | 1322.557000 | 905.1038 |
| 806.5 | 272.9 |  | 3037.369000 | 1205.0770 |
| 830.0 | 182.0 |  | 3010.069000 | 1070.4970 |
| 634 ± 86 | 231 ± 15 |  | 2059 ± 409 | 1109 ± 64 |
| Obs. These are also the individual values plotted in the linear regression showed in **Figure 5B** | | | | |

| **Figure 5D** | | | | | | |  |
| --- | --- | --- | --- | --- | --- | --- | --- |
| %LTC4+ granuloma eosinophils | | | |  | Lipid bodies/granuloma eosinophil | | |
| PGD2 | Non-stimulated | S. mansoni antigen | S. mansoni antigen + CAY |  | Non-stimulated | S. mansoni antigen | S. mansoni antigen + CAY |
| 52.0 | 0.0 | 40.0 | 23.0 |  | 11.18 | 19.40 | 16.80 |
| 76.6 | 40.0 | 73.9 | 43.3 |  | 17.42 | 19.90 | 18.60 |
| 70.0 | 46.6 | 67.6 | 46.6 |  | 9.77 | 23.05 | 19.48 |
| 75.0 | 13.3 | 80.0 | 27.0 |  |  |  |  |
|  |  | 88.9 | 30.0 |  |  |  |  |
|  |  | 66.6 | 43.3 |  |  |  |  |
| 68.4 ± 5.6 | 24.9 ± 11.0 | 69.5 ± 6.8 | 35.5 ± 4.1 |  | 12.79 ± 2.3 | 20.78 ± 1.1 | 18.29 ± 0.8 |

| **Figure 6A** | | |
| --- | --- | --- |
| Hepatic PGD2 | | |
| Non Infected | Infected | Inf + MK571 |
| 138.705200 | 163.862800 | 263.292400 |
| 71.703110 | 102.652100 | 181.146000 |
| 58.345600 | 122.267100 | 127.251500 |
| 10.711300 | 275.327000 | 125.405600 |
|  | 82.402060 | 184.696000 |
|  | 183.794800 | 155.713600 |
|  | 111.515200 | 86.380920 |
| 69.9 ± 26.4 | 148.8 ± 24.9 | 160.6 ± 21.5 |

| **Figure 6B** | | | | | |  |
| --- | --- | --- | --- | --- | --- | --- |
| Blood eosinophils | | | Eosinophils/granuloma section | | |  |
| Non Infected | Infected | Inf + MK571 |  | Infected | Inf + MK571 | |
| 0.0 | 1.4 | 1.5 |  | 280.6600 | 369.5000 | |
| 0.5 | 0.8 | 0.8 |  | 208.8333 | 411.0000 | |
| 0.2 | 1.7 | 1.3 |  | 375.7500 | 414.0000 | |
| 0.6 | 1.0 | 0.5 |  | 208.1667 | 161.5000 | |
|  | 0.4 | 0.6 |  | 198.5000 | 250.3333 | |
|  | 1.0 | 0.7 |  | 286.3333 | 251.1667 | |
|  | 1.5 | 1.2 |  | 177.1667 | 286.5000 | |
| 0.3 ± 0.1 | 1.1 ± 0.2 | 0.9 ± 0.1 |  | 247.9 ± 26.5 | 306.3 ± 35.9 | |

| **Figure 6C** | | | | | | |
| --- | --- | --- | --- | --- | --- | --- |
| Hepatic TGF-beta | | |  | Hepatic IL-13 | | |
| Non Infected | Infected | Inf + MK571 |  | Non Infected | Infected | Inf + MK571 |
| 0.000000 | 90.991970 | 209.328000 |  | 0.901916 | 7.169308 | 14.468790 |
| 62.392080 | 203.819900 | 176.947300 |  | 0.5194464 | 4.998277 | 11.901200 |
| 40.098900 | 93.669900 | 179.507000 |  | 0.901916 | 3.328974 | 11.420050 |
| 0.000000 | 40.141400 | 194.510800 |  | 0.000000 | 2.486941 | 2.698194 |
|  | 129.818400 | 217.559700 |  |  | 8.015668 | 13.654470 |
|  | 172.107800 | 134.501500 |  |  | 1.286448 | 3.765447 |
|  | 63.114410 | 290.032500 |  |  | 1.450176 | 14.478220 |
| 25.6 ± 15.5 | 113.4 ± 22.1 | 200.3 ± 18.1 |  | 0.6 ± 0.2 | 4.1 ± 1.0 | 10.3 ± 1.9 |

| **Figure 6D** | |
| --- | --- |
| Number of egg+ granulomas | |
| Infected | Inf + MK571 |
| 3.9 | 3.3 |
| 3.3 | 0.8 |
| 0.4 | 2.2 |
| 1.0 | 1.4 |
| 1.7 | 1.8 |
| 1.5 | 3.0 |
| 3.3 | 0.4 |
| 2.2 ± 0.5 | 1.8 ± 0.4 |

| **S1 Fig** | | | | | | | | |
| --- | --- | --- | --- | --- | --- | --- | --- | --- |
| Serum IL-5 | | | |  | Serum IL-13 | | | |
| Non Infected | Infected | NonInf+HQL | Inf + HQL |  | Non Infected | Infected | NonInf+HQL | Inf + HQL |
| 35.62401 | 109.32660 | 5.36598 | 87.33250 |  | 16.300590 | 410.8322 | 9.124088 | 362.962200 |
| 4.30128 | 46.53630 | 9.32660 | 49.52367 |  | 25.112760 | 363.8250 | 6.636086 | 375.247500 |
| 9.36522 | 54.42370 | 4.63260 | 74.75920 |  | 15.603010 | 365.8231 | 45.179700 | 381.643900 |
| 5.33257 | 27.95630 | 6.32660 | 31.64520 |  | 5.160988 | 360.2039 | 4.184750 | 348.738600 |
| 33.01987 | 47.36266 | 45.32659 | 27.82560 |  | 13.872450 | 359.9113 | 24.927680 | 356.958500 |
| 48.89650 |  |  |  |  | 29.054500 |  |  |  |
| 22.8 ± 7.7 | 57.1 ± 13.8 | 14.2 ± 7.8 | 54.2 ± 11.7 |  | 17.5 ± 3.5 | 372.1 ± 9.7 | 18.0 ± 7.7 | 365.1 ± 6.0 |

| **cont. S1 Fig** | | | |
| --- | --- | --- | --- |
| Bone marrow eosinophils | | | |
| Non Infected | Infected | NonInf+HQL | Inf + HQL |
| 0.810000 | 1.203000 | 0.251000 | 2.334500 |
| 0.744000 | 1.450000 | 0.090500 | 0.544500 |
| 0.384000 | 2.907000 | 0.210000 | 0.962500 |
| 0.407500 | 0.680000 | 0.116000 | 2.673000 |
| 0.346000 | 0.469000 | 0.415000 | 2.505000 |
| 0.198000 |  |  |  |
| 0.5 ± 0.1 | 1.3 ± 0.4 | 0.2 ± 0.1 | 1.8 ± 0.4 |

| **S2 Fig A** | | | | |
| --- | --- | --- | --- | --- |
| Peritoneal PGD2 | |  | Hepatic PGD2 | |
| Non Infected | Infected |  | Non Infected | Infected |
| 32.365260 | 103.021400 |  | 0.000000 | 121.326600 |
| 51.236590 | 88.326580 |  | 63.023150 | 290.326900 |
| 82.321500 | 189.325700 |  | 34.032570 | 61.032560 |
|  | 152.020000 |  |  | 85.236500 |
| 55.31 ± 14.6 | 133.2 ± 23.1 |  | 32.4 ± 18.2 | 139.5 ± 51.8 |

| **S2 Fig B** | |
| --- | --- |
| Number of egg+ granulomas | |
| Non Infected | Infected |
| 0.0 | 0.05 |
| 0.0 | 0.50 |
| 0.0 | 1.15 |
|  | 0.25 |
| 0.0 ± 0.0 | 0.5 ± 0.2 |

| **S2 Fig C** | | | | |
| --- | --- | --- | --- | --- |
| Peritoneal eosinophils | |  | Hepatic eosinophils | |
| Non Infected | Infected |  | Non Infected | Infected |
| 0.085000 | 0.020000 |  | 0.0 | 0.5 |
| 0.120000 | 0.000000 |  | 0.0 | 0.2 |
| 0.110000 | 0.210000 |  | 0.0 | 0.5 |
|  | 0.090000 |  |  | 0.0 |
| 0.1 ± 0.01 | 0.1 ± 0.05 |  | 0.0 ± 0.0 | 0.3 ± 0.1 |

| **cont. S2 Fig C** | | | | |
| --- | --- | --- | --- | --- |
| Blood eosinophils | |  | Bone marrow eosinophils | |
| Non Infected | Infected |  | Non Infected | Infected |
| 0.150000 | 0.100000 |  | 0.550000 | 1.950000 |
| 0.080000 | 0.550000 |  | 1.250000 | 2.450000 |
| 0.050000 | 0.400000 |  | 0.350000 | 2.800000 |
|  | 0.150000 |  |  | 3.700000 |
| 0.1 ± 0.02 | 0.3 ± 0.11 |  | 0.7 ± 0.3 | 2.7 ± 0.4 |

| **S2 Fig D** | | | | |
| --- | --- | --- | --- | --- |
| Peritoneal LTC4 | |  | Hepatic LTC4 | |
| 1.562400 | 2.454000 |  | 45.021480 | 0.000000 |
| 0.512400 | 0.898500 |  | 0.000000 | 0.000000 |
| 0.925400 | 1.021400 |  | 100.325000 | 32.021500 |
|  | 1.302140 |  |  | 55.721700 |
| 1.0 ± 0.3 | 1.4 ± 0.4 |  | 48.5 ± 29.0 | 21.9 ± 13.6 |

| **S2 Fig E** (in vitro) | |
| --- | --- |
| PGD2 from stellate cells | |
| Non Infected | Infected |
| n.d. | 18.15 |
| n.d. | 29.20 |
|  | 23.7 ± 5.2 |

.

| **S3 Fig A and S3Fig B** | | | | | | |
| --- | --- | --- | --- | --- | --- | --- |
| Peritoneal mononuclear cells | | | |  | Lipid bodies/peritoneal mono cells | |
| Non Infected | Infected | NonInf+ CAY | Inf + CAY |  | Infected | Inf + CAY |
| 9.3 | 31.3 | 4.6 | 38.0 |  | 0.88 | 0.840 |
| 8.0 | 34.1 | 6.1 | 34.2 |  | 0.90 | 0.440 |
| 4.9 | 34.3 | 5.9 | 14.1 |  | 0.82 | 0.620 |
| 6.5 | 36.1 | 9.9 | 37.0 |  | 0.82 | 0.540 |
| 5.6 | 45.5 | 13.7 | 30.4 |  | 0.68 |  |
| 9.9 | 37.3 | 11.1 | 52.7 |  |  |  |
| 5.8 | 37.8 |  |  |  |  |  |
|  | 32.3 |  |  |  |  |  |
| 7.1 ± 0.7 | 36.1 ± 1.6 | 8.6 ± 1.5 | 34.4 ± 5.1 |  | 0.8 ± 0.04 | 0.6 ± 0.09 |

| **S4 Fig** (in vitro) | |
| --- | --- |
| PGD2 from granuloma eosinophils | |
| Non stimulated | S. mansoni antigen |
| 16.60 | 33.30 |
| 10.00 | 26.30 |
| 28.00 | 46.30 |
| 18.2 ± 5.2 | 35.3 ± 5.9 |
