# Supplementary material for: Endogenous PGD2 acting on DP2 receptor counter regulates Schistosoma mansoni infection-driven hepatic granulomatous fibrosis
Source: PLoS Pathog. 2024 Aug 22;20(8):e1011812. doi: 10.1371/journal.ppat.1011812 (PMC11386465; doi:10.1371/journal.ppat.1011812)
Supplement: Attachment — Submitted filename: reviewers.docx [file ppat.1011812.s008.docx]

**Authors’ answers to reviewers**

**Part I:**

**Reviewer #1**: The authors of this study demonstrated that the activation of PGD2 and its receptor may play a protective role in liver fibrosis following S. mansoni infection. Key findings from this research reveal that inhibiting PGD2 synthesis using HQL-79 and blocking the DP2 receptor with an antagonist resulted in elevated levels of TGFb and IL-13 in the liver, which are believed to promote fibrogenesis. An intriguing discovery in this study is that treatment with CAY10471, a known DP2 receptor blocker, led to a decrease in the number of eosinophils in the liver sections of S. mansoni-infected mice. Additionally, CAY10471 treatment also promoted a fibrotic reaction within the hepatic tissue of infected mice. The authors further established a link between LTC4 production and liver fibrosis, identifying eosinophils as the source of LTC4. Both in vitro and in vivo evidence demonstrated reduced levels of LTC4 when mice or eosinophils were treated with CAY10471. The conclusion that PGD2 activates DP2 receptors, stimulating cysLTs production by eosinophils, and subsequently inhibiting liver fibrosis is both interesting and significant. The paper is well-written and deserves publication.

**Reviewer #3**: The liver is the main cause of health problems associated with schistosomiasis. A potential role for PGD2 in promoting liver fibrosis has been demonstrated in vitro. Thus, it was suggested that PGD2 inhibition would benefit people with schistosomiasis. However, there is no direct evidence in a schistosomiasis mouse model.
The Authors describe the effect of inhibiting the production or action of PGD2 in a mouse model of schistosomiasis. They also investigated how this PGD2 effect is linked to eosinophils and to LTC4. Surprisingly the data indicate that PGD2 protects the liver from fibrosis rather than causing it. Thus, its inhibition might aggravate liver failure and not benefit patients with schistosomiasis.

The paper's background is well reasoned and brings the rationale to assess the role of PGD2 in liver fibrosis in Schistosoma mansoni infected mice. The subject is important. The research is exacting, well-executed and well-documented.

However, the manuscript is too complex. I would have suggested that they go more in depth on one of the various directions: which cells besides eosinophils are responsible for the PGD2 pro-fibrotic or anti-fibrotic effects, by which mechanisms in vitro and in vivo is this carried out, etc. For example, the Authors mention the important role of mast cells as main cells producing PGD2 and being present in the liver granulomas but fail to study this.

We thank both authors for recognizing the relevance of the study and for the suggestions that have definitively improved it. The revised *ms* is now more focused on establishing the protective role of endogenous PGD_2_ activating DP2 receptors on schistosomal fibrosis by a mechanism dependent on subsequent LTC_4_ synthesis/action; and therefore, cautions against therapies targeting these lipidic molecules.

Because the revised version investigates in greater depth the endogenous anti-fibrogenic role of endogenous PGD_2_/DP2 in schistosomiasis, a thorough identification of either PGD_2_ cell sources or PGD_2_ cell targets was not our main goal here. Nonetheless, we now did clarify throughout the *ms* that stellate myofibroblasts of hepatic granulomas appear to be the main cell source of the initial anti-fibrogenic PGD_2_ (**see new S2 Fig** and **S4 Fig**), while DP2-expressing eosinophils seem to represent the main PGD_2_ cell target. Future studies were already designed to establish the complete cellular component of the PGD_2_-driven protective functions uncovered here, mainly focusing on stellate cells and eosinophils, but also analyzing the putative role of mast cells. For the sake of linearity, our off-focus paragraph on mast cells in the original *ms* version was removed from the revised version.

As suggested by **reviewer #3**, we chose to go more in-depth in only one specific direction: the evaluation of the potential anti-fibrogenic role of endogenous LTC_4_ in *S. mansoni* infection (**see new** **Fig 6**). Finally, to ease the complexity of the original version of the *ms*, we have trimmed it and elaborated a schematic summary of a proposed mechanistic model based on our main findings (**see new Fig 7**).

**Part II:**

**Reviewer #1**: However, there are a few weak points that need to be addressed:

**1.** It is important to discuss the potential differences in the consequences of DP2 deficiency/blockade on eosinophils in various models. For example, in an allergic inflammatory model of asthma, DP2/CRTH2 knockout mice displayed enhanced eosinophil recruitment into the lung compared to wild-type littermate mice (The Journal of Immunology, 175(4), 2056-2060). Similarly, in cases of renal fibrosis and lung fibrosis, DP2/CRTH2 has been shown to have profibrotic roles (American Journal of Respiratory Cell and Molecular Biology, 67(2), 201-214).

Done.

We agree that this information is important to the rationale of our study, therefore we have added both citations brought by the reviewer, as follows:

- As new reference number 62, in “Results and Discussion section” (line 415 ): “More than simple ratifying HQL-79 data, and in clear contrast to data showing enhanced allergic pulmonary eosinophilia in DP2 deficient mice [62], CAY10471-driven findings upgraded DP2 to one of the key eosinophilotactic receptors in schistosomiasis, like CCR3 [50].”

- As new reference number 30, in the “Introduction” section (line139) reads “In the lung, while studies on non-asthmatic models of pulmonary fibrosis acknowledge PGD_2_ itself (and its DP2 receptor) as either beneficial [26-29] or deleterious [30] signal against fibrotic development; in the asthmatic lung, however, PGD_2_ displays undisputable pro-fibrogenic impact [31,32].”

**2.** It would be beneficial to include in vivo models with DP2/CRTH2 conditional deletion in eosinophils to further elucidate how PGD2/DP2 contributes to liver pathology during S. mansoni infection.

Yes, we agree that it would be enlightening.

However, since these animals are not commercially available and establishing these mouse strains is not feasible for the current *ms*, we have directed our efforts to characterize the role of eosinophil-derived cysteinyl leukotrienes as a CysLT1 agonists in schistosomiasis-induced hepatic fibrosis (see description bellow and **new Fig 6**). While investigating the role of eosinophils in *in vivo* PGD_2_/DP2 anti-fibrogenic effect was not the primary goal of the present study, we have nonetheless added new supplemental data (**S2 Fig**) showing that in an early time point during hepatic granuloma development (6 wpi) prior eosinophil arrival, the levels of hepatic PGD_2_ are already elevated with no detectable LTC_4_. This data uncouples the initial production of PGD_2_ from eosinophils and reinforces infection-induced LTC_4_ synthesis as a phenomenon that depends on eosinophil presence. New **S2 Fig** also shows that 6 wpi-related hepatic stellate cells (granuloma’s myofibroblasts) produce PGD_2_ under *S. mansoni*-driven stimulation.

**3.** If establishing an eosinophil-specific knockout of DP2 is not feasible, the authors should consider supplementing their data to further support the contribution of eosinophils in alleviating liver fibrosis caused by S. mansoni infection, specifically through DP2 and LTC4 production. One possible approach could be to investigate the effects of depleting eosinophils. By depleting eosinophils in an animal model, the authors could observe the impact on liver fibrosis progression and evaluate whether the absence of eosinophils alters the protective effects mediated by the PGD2-DP2-LTC4. This additional data would strengthen the understanding of the role of eosinophils in the context of liver fibrosis and help solidify the conclusions drawn in the study.

We thank the reviewer for the suggestion on how to investigate the role of eosinophils in the PGD2-DP2-LTC4 axis regulating liver fibrosis. Inasmuch as eosinophil depletion experiments are part of our roadmap to understand the pathogenesis of hepatic granulomas in *S. mansoni* infections, those studies became outside of the scope of the current manuscript when we decided to further the understanding of the anti-fibrogenic molecular role of endogenous DP2 activation by PGD_2_. We, however, reinforced the role of PGD_2_/DP2-stimulated eosinophils as the producer of anti-fibrogenic LTC_4_ during *S. mansoni* infection-induced hepatic granulomatous fibrosis through in vivo and in vitro studies that clarified the kinetics of LTC4 production and the eosinophil as its cellular source (**S2 Fig** and **Fig 6**).

We venture to say that the forthcoming studies will include not only eosinophil depletion, but other experimental approaches to investigate the role of eosinophils, mast cells, and stellate cells in liver granulomas.

**Reviewer #3**: To strengthen the paper, additional experiments could be conducted on:
-- Eosinophil deficient mice in which PGD2 activity is inhibited;

-- PGD2 knock-out mice;

-- In mice in which pharmacological blockage of LOX5 is achieved.

We appreciate the suggestion made by the reviewer and decided to further our studies focusing on the third suggestion. Therefore, we conducted an experiment to study the potential role of 5-LO-derived metabolite LTC_4_, which we initially found enhanced and inversely correlated with the magnitude of granulomatous fibrosis in *S. mansoni*-infected liver. After fully consideration of how to address this key issue, we have decided for a more specific approach than the one suggested by the reviewer. Instead of inhibiting the upstream enzyme 5-LO – that would nonspecifically inhibit the synthesis of various 5-LO-driven bioactive lipids besides LTC_4_ – we employed a specific antagonist for CysLT1 receptors. As can be observed in **new Fig 6**, the treatment with MK571 promoted a similar increase in infection-induced production of TFG-β and IL-13 within the granulomatous liver, but distinctly from HQL-79 and CAY10471, it did not affect local PGD_2_ production or eosinophil amounts. Collectively, this data unveils that in a *S. mansoni*-driven eosinophil-enriched environment under PGD_2_ stimulation, LTC_4_ acting on its receptors enhances the pro-fibrinogenic profile of hepatic granulomatous reaction.

**Part III:**

**Reviewer #1**: (No Response)

**Reviewer #3**: The manuscript is too heavy and complex. It is not fluid reading. The itemization -- 1,2,3,4 -- is too much. It should be re-written and shortened to make it easier to read and understand.

As requested, we have worked to make the *ms* easier to read. Although it may not have a significant impact in the length of the manuscript due to the inclusion of new data, we have cut about 700 words from the initial version, removing paragraphs to improve the fluidity of the *ms*. We have also eliminated several “itemizations” throughout the text. To make it easier to read we also included a schematic figure detailing our main findings (new **Fig 7**).
