# Supplementary material for: Endogenous PGD2 acting on DP2 receptor counter regulates Schistosoma mansoni infection-driven hepatic granulomatous fibrosis
Source: PLoS Pathog. 2024 Aug 22;20(8):e1011812. doi: 10.1371/journal.ppat.1011812 (PMC11386465; doi:10.1371/journal.ppat.1011812)
Supplement: Attachment — Submitted filename: Response to reviewers R2.docx [file ppat.1011812.s009.docx]

Authors’ Answers to Questions

**Part I - Summary**
Please use this section to discuss strengths/weaknesses of study, novelty/significance, general execution and scholarship.

Reviewer #1: The data from additional experiments in the revised manuscript addressed my concerns despite the fact that the eosinophil specific genetic mouse models are not available. The descriptions to put less emphasis on eosinophil specific roles as shown in line 107 to 113, Page 71 were appropriate.

Reviewer #4: The authors demonstrated that PGD2 by activating DP2 receptors stimulates cysLTs production by eosinophils, while endogenously down-regulates the hepatic fibrogenic process of S. mansoni granulomatous reaction – an in vivo protective function which demands caution in the future therapeutic attempts in targeting PGD 2/DP2 in schistosomiasis. The authors inhibited the production or action of PGD2, and antagonized CysLT1 in a mouse model of schistosomiasis. Furthermore, in vitro experiments provided further evidence for the presented conclusions.
The study is well conducted and the data presented represent a valuable contribution to research into schistosomiasis.

We thank both reviewers for the positive comments and their impactful role in improving the manuscript.

**Part II – Major Issues: Key Experiments Required for Acceptance**
Please use this section to detail the key new experiments or modifications of existing experiments that should be absolutely required to validate study conclusions.

Generally, there should be no more than 3 such required experiments or major modifications for a "Major Revision" recommendation. If more than 3 experiments are necessary to validate the study conclusions, then you are encouraged to recommend "Reject".

Reviewer #1: none

Reviewer #4: Figures 2 A and B: Hepatic Hydroxyproline levels should be checked carefully. There seems to be an error in the presented data. 100-180 µg/g hydroxyproline can be found in the liver of healthy C57BL/6 mice while 300-400µg hydroxyproline per g liver tissue was detected in mice infected with S. mansoni for 9 weeks (PMID: 37990129).

We thank Reviewer #4 for pointing out the discrepancy between the data included in our manuscript and the literature. We were unfamiliar with this particular assay and focused only on the qualitative aspects without noticing that our values were off by a lot. It turns out that we failed to consider the dilution factor of the assay reaction. When this dilution factor corrects samples, their calculated values are within the expected range. We are disconcerted by the mistake and glad that it was caught by the reviewer before it was included in the scientific record. Figures 2A and 2B, and Figure 3C were corrected with the actual level of OH-proline/g of hepatic tissue.

**Part III – Minor Issues: Editorial and Data Presentation Modifications**
Please use this section for editorial suggestions as well as relatively minor modifications of existing data that would enhance clarity.

Reviewer #1: (No Response)

Reviewer #4: 1. Complicated wording makes understanding difficult:
e.g. Lines 298-302: First, distinct from the eosinophilic reaction impairment observed in DP1 receptor-deficient model [19] , HQL-79-unveiled PGD2 role in S. mansoni infection driven eosinophilia does not correspond to an inevitable consequence of a large inhibition of S. mansoni parasitism or overall lack of subsequent type 2 immune response.

Thanks for the suggestion, we changed the sentence. New phrasing now reads: “First, HQL-79-unveiled PGD_2_ role in eosinophilia induced by *S. mansoni* infection is neither a consequence of *S. mansoni* parasitism inhibition nor a failure to mount the subsequent type 2 immune response. Therefore, the mechanism involved is distinct from the observed in DP1 receptor-deficient model [19].”

2. Perhaps separating the results and discussion into two separate sections would make the manuscript easier to understand.

We appreciate the suggestion. However, we arrived at the current format for results and discussion after considering having them in separate sections. The final text was more concise and clearer when the data and its implications were described as a single section rather than having a long-winded discussion section that rehashed the results.
