# Supplementary material for: Endogenous PGD2 acting on DP2 receptor counter regulates Schistosoma mansoni infection-driven hepatic granulomatous fibrosis
Source: PLoS Pathog. 2024 Aug 22;20(8):e1011812. doi: 10.1371/journal.ppat.1011812 (PMC11386465; doi:10.1371/journal.ppat.1011812)
Supplement: Attachment — Submitted filename: Reviewer Comments.docx [file ppat.1011812.s007.docx]

Reviewer comments

In this research, authors clarified that PGD2 by activating DP2 receptors stimulates cysLTs production by eosinophils, while endogenously down-regulates the hepatic fibrogenic process of *S. mansoni* granulomatous reaction. I*n vivo* protective function which demands caution in the future therapeutic attempts in targeting PGD2/DP2 in schistosomiasis. However, the evidence is not sufficient, and the author overinterprets the experimental results, leading to the deviation of the conclusion.

**Major review**

1. In the abstract, “An ample body of data supports the role of *S. mansoni*-driven DP2-mediated activation of eosinophils as a key down-regulator of schistosomiasis-induced liver fibrosis,” The authors tend to suggest that eosinophils down-regulate hepatic fibrosis, although this conclusion is controversial. The evidence for this conclusion is not strong enough.
2. In the abstract, the authors think that “peritoneal eosinophils were identified as the only cells producing LTC4 in PGD2-mediated *S. mansoni*-induced infection”. In Fig 4C, it is difficult to accurately distinguish neutrophils, basophils, and eosinophils by morphology alone. It is recommended to detect the expression-specific marker molecules on the cells to identify which cells in granuloma express LTC4 using flow cytometry.
3. In the abstract, one of the most important conclusions: “our findings uncover that PGD2 by activating DP2 receptors stimulates cysLTs production by eosinophils, while endogenously down-regulates the hepatic fibrogenic process of *S. mansoni* granulomatous reaction”，actually, The phenomenon of eosinophils releasing PGD2 and participating in inflammatory responses through DP1 and DP2 receptors is widely present in various diseases, The article lacks evidence of the relationship between cysLTs production and liver fibrosis caused by *S. mansoni*.
4. After administrated of HQL-79 and CAY10471, eosinophils were destroyed, and liver fibrosis was aggravated in mice infected with *S. mansoni*. However, no experiments have proven a cause-and-effect relationship. It's unclear whether it reduces LTC4 and promotes fibrosis by destroying eosinophils, or promotes liver fibrosis by promoting TGF-β, IL-13 secretion. It is unclear whether HQL-79 and CAY10471 have other effects than inhibitors of the PGD2/DP2 pathway.
5. If the authors want to make it clear that inhibition of PGD2/DP2 can destroy eosinophils, you can add an experiment. Administration activator of PGD2/DP2 to observe the changes in liver fibrosis. It could confirm that PGD2/DP2 is a potential target for the treatment of liver fibrosis.

**Minor review**

1. Line 68 “However, our data indicates that PGD2 protects the liver from fibrosis”. The evidence for this claim is insufficient, and PDG2 activators are also needed to observe that liver fibrosis in schistosomiasis is indeed reduced.
2. Line 308, “First, …” and Line 406,” may depend on an eosinophil related three-component mechanism,” which are overexplained.
3. The method of calculating egg area needs to be described carefully. The area of granuloma is related to the number of eggs deposited in liver tissue, and the area of a single egg granuloma should be selected for comparison of granuloma area in different groups.
4. From Line 592 to 594, for the separation of eosinophils, it is recommended to use the method of tissue digestion and immunomagnetic bead sorting to isolate the cells with higher purity.
